# 

**Published:** 1888-02

**Authors:** 


					﻿v ‘ FEBRUARY, 188E
OLD SERIES
VoU XXV
4* 1855 4
NEW SERIES
* Vol. IV.-No. 12.
THtih®
fflEDItSE«OS

Jpp by™ W.F.WESTMOREL?AND.I^
H.VM.M1LLER.M.D.LL.D.1
ip	JAMES A.GRAY. MJJ.
PUBLISHED BY JAMES P.HARRISON&CG3
Atlanta.ga.
SUBSCRIPTION: S2.5O A YEAR.
				

